# Recommended guidelines concerning artificial intelligence and health equity: a scoping review

**DOI:** 10.1093/haschl/qxag236

**Published:** 2026-10-08

**Authors:** Caroline R Efird, Claudia Park, Parth Mishra, Angela Barr, Andrew J Twinamatsiko, Christopher J King

**Affiliations:** Department of Health Management and Policy, School of Health, Georgetown University, Washington, DC 20057, United States; Department of Health Management and Policy, School of Health, Georgetown University, Washington, DC 20057, United States; Department of Health Management and Policy, School of Health, Georgetown University, Washington, DC 20057, United States; William M. Anderson Library, West Shore Community College, Scottsville, MI 49454, United States; O’Neill Institute for National and Global Health Law, Georgetown University Law Center, Washington, DC 20001, United States; Department of Health Management and Policy, School of Health, Georgetown University, Washington, DC 20057, United States

**Keywords:** health equity, health disparities, artificial intelligence, AI, health services research, healthcare systems

## Abstract

**Introduction:**

The use of artificial intelligence (AI) technologies in healthcare has potential to improve patient care, expand access, and optimize efficiency. Yet, a growing body of evidence warns of health risks for marginalized and minoritized populations as a result of algorithmic bias.

**Methods:**

To synthesize leading scholars’ recommendations for equitable AI implementation in healthcare settings and health services research, we conducted a scoping review of current literature.

**Results:**

We identified and analyzed 166 relevant, peer-reviewed articles. Most articles (70%) originated in the United States. Only 13% provided a specific definition of “health equity.” Included manuscripts generally emphasized that ethical human oversight must accompany AI-use. The engagement of diverse groups of stakeholders during model development, implementation, and evaluation is also paramount. Patient and provider education on AI's capabilities is also critical for building trust and equitable delivery of care.

**Conclusion:**

To enhance the field's ability to implement AI in ways that address health disparities and inequities, future research needs to amplify discipline-specific expertise and more global perspectives. By explicitly prioritizing health equity, transparency, inclusion, and education, there is potential to meaningfully regulate AI in healthcare to redress systemic inequities in care and advance health and well-being in populations throughout the world.

## Introduction

Various forms of artificial intelligence (AI) are rapidly shaping how health services are organized, delivered, and financed. AI technologies are powerful tools with potential to improve healthcare delivery for patients, providers, and healthcare systems.^1^ Yet, a growing body of evidence warns of potential risks for marginalized and minoritized populations as a result of algorithmic bias.^2-4^ Marginalized and minoritized groups experience various forms of discrimination or vulnerability as a result of societal, political, and organizational conditions that differentially distribute power and resources.^5^ The lack of transparency and stewardship concerning AI use is concerning because marginalized populations are likely to experience the greatest health risk as a result.^6^

To effectively promote health and well-being, healthcare systems and health scientists must consider the risks of AI in exacerbating or sustaining disparities in health outcomes. The World Health Organization (WHO) defines health equity as “the absence of unfair, avoidable, or remediable differences among groups of people, whether those groups are defined socially, economically, demographically, or geographically or by other dimensions of inequality (eg, sex, gender, ethnicity, disability, or sexual orientation).”^5^ Appropriate measures are therefore necessary to ensure that AI and associated technologies do not adversely affect access and quality of care rendered to marginalized and minoritized populations. Accordingly, we seek to synthesize leading scholars’ suggested guidelines for equitable use of AI in healthcare settings and health services research.

Our research aims are 2-fold: (1) identify peer-reviewed commentaries, literature reviews, scoping reviews, analytic essays, opinion articles, editorials, and reports that discuss the role of AI as it relates to equity in healthcare systems, healthcare practice, and health services research and (2) compare equitable uses of AI in healthcare systems, healthcare practice, and health services research. Our goal is to summarize current literature, identify gaps in the existing knowledge base, and inform directions for future research and practice at the intersection of AI and health equity. We intend to build upon prior, related syntheses^4,6,7^ by offering a broader and more recent scope of the literature through the collection of global and cross-disciplinary perspectives spanning health-equity focused AI-use in clinical, research, and healthcare practice-based settings.

## Methods

### Protocol

Our review protocol is based on Arskey and O’Malley's^8^ scoping review framework and Tricco and colleagues’^9^ best practices for scoping reviews. We registered our protocol on Open Science Framework on December 22, 2024 (https://osf.io/84sf2/).

### Eligibility criteria

We sought peer-reviewed commentaries, literature reviews, scoping reviews, analytic essays, opinion articles, and editorials, which broadly discuss the role of artificial intelligence, as it relates to health equity in healthcare settings, healthcare systems, and health services research.

### Data sources and searches

The review team performed searches between January 14-22, 2025 in the following databases: PubMed (January 1, 2014 to January 22, 2025, NIH.gov), Academic Search Premiere (1914 to January 14, 2025, EBSCOhost), Web of Science (January 1, 2014 to January 15, 2025, Clarivate), and Google Scholar (2014-2025, Google). No limits were applied to the search results unless noted. With input from the other members of the review team, an experienced health sciences librarian, AB, designed and executed the primary search in the PubMed database (see Appendix 1). The PubMed search was then translated for use in the other databases. The search strings primarily utilized controlled vocabulary and, to a lesser extent, keywords present in the title/abstract of the citations to represent the search concepts of “artificial intelligence” and “health equity/inequity.” AB conducted the initial database search and de-duplication processes. Following de-duplication, 860 citations were initially included for screening (see Figure 1).

**Figure 1.**
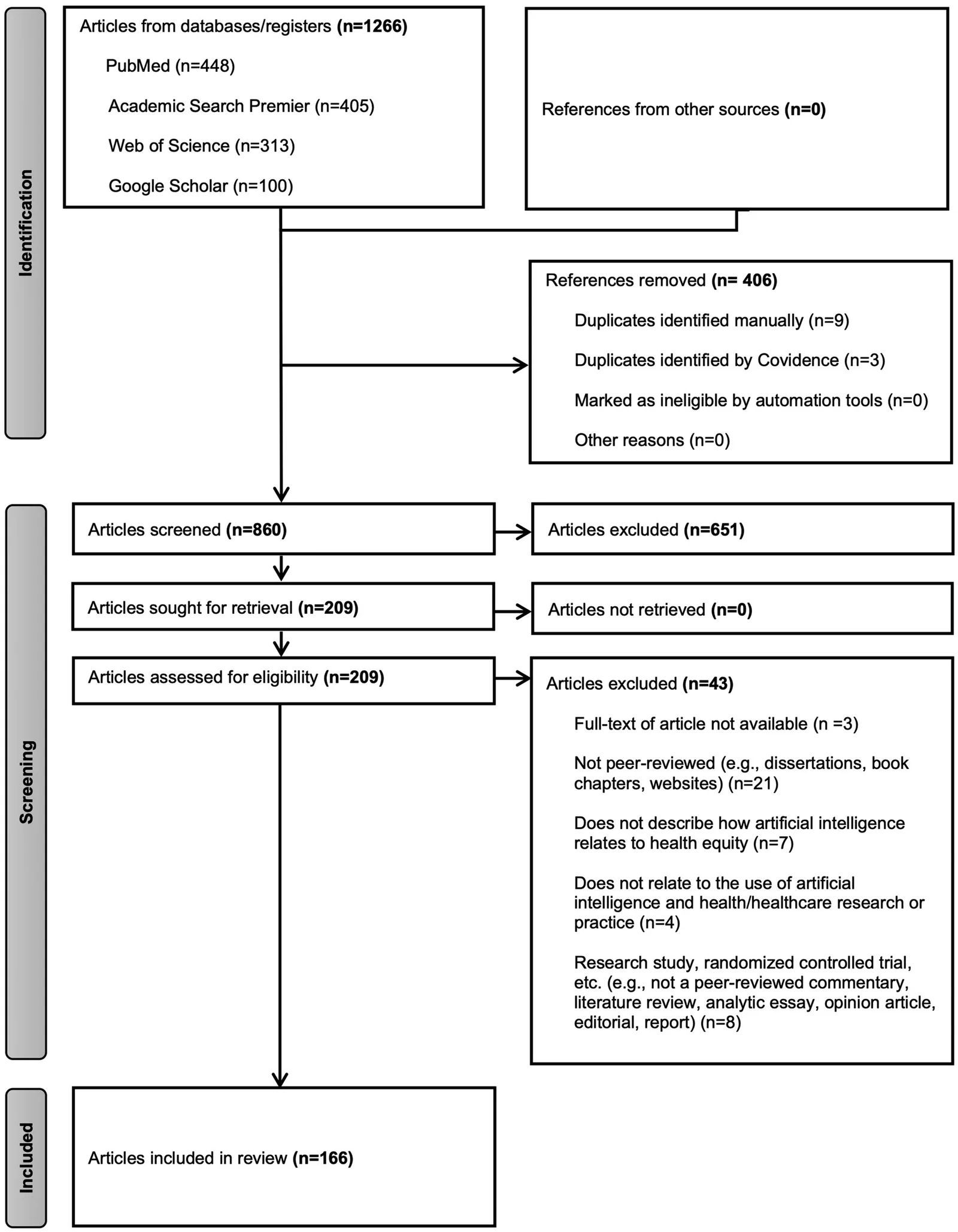
Preferred Reporting Items for Systematic Reviews and Meta-Analyses Extension for Scoping Reviews (PRIMSA-ScR) diagram for health equity and artificial intelligence review.

### Data collection and synthesis

CE and 3 research assistants conducted duplicate, blinded, and independent reviews and decisions on inclusion or exclusion of abstracts of 860 articles in Covidence software. Conflicts were resolved by CE. A second round of full text screening (*n* = 209) was conducted by CP, PM, and another research assistant. Conflicts were resolved by CE. See the PRISMA-ScR^10^ flow diagram for details on the screening process (Figure 1). All authors reviewed and approved the final list of articles (*n* = 166) before CE, CP, and PM began the data extraction process.

We extracted the following information from each publication:

- Title
- Author(s)
- Year of publication
- Location of institutional affiliation of first author
- Country or region of focus (if applicable)
- Type of peer-reviewed publication (eg, commentary, literature review, analytic essay, opinion article, editorial, and report)
- Primary audience of the journal
- Aims/purpose
- Author(s)’ definition/conceptualization of health equity
- Type of artificial intelligence addressed (eg, natural language processing, machine learning, large language models, deep learning)
- Focus area (eg, healthcare systems, healthcare settings, health services research)
- Specific population addressed (if applicable)
- Specific health outcome addressed (if applicable)
- Key findings/suggestions that relate to equitable uses of artificial intelligence in healthcare systems, clinical settings, health services research, and healthcare practice

To facilitate interpretation of the qualitative data we extracted from each manuscript, we developed analytic memos to classify and synthesize included manuscripts’ strategies for ethical and equitable AI use. Additionally, we created data matrices and analytic memos to identify potential patterns in the articles that presented a definition of health equity. CE, AT, and CK met to discuss data synthesis and presentation of Results.

## Results

Characteristics of the 166 included publications are detailed in Table 1. Manuscripts originated in the United States (*n* = 116), United Kingdom (*n* = 10), Canada (*n* = 9), Germany (*n* = 5), India (*n* = 3), Philippines (*n* = 3), China (*n* = 2), Switzerland (*n* = 2), Australia (*n* = 1), Austria (*n* = 1), Bangladesh (*n* = 1), Finland (*n* = 1), Gabon (*n* = 1), Iran (*n* = 1), Israel (*n* = 1), Luxembourg (*n* = 1), Netherlands (*n* = 1), Norway (*n* = 1), Poland (*n* = 1), Spain (*n* = 1), Sri Lanka (*n* = 1), Sweden (*n* = 1), Thailand (*n* = 1), and Türkiye (*n* = 1). Most manuscripts were geared toward a general or global audience (*n* = 139), with some focusing on the following specific countries or regions: United States (*n* = 16), Europe (*n* = 3), low- and middle-income countries (*n* = 2), India (*n* = 1), Spain (*n* = 1), South Korea (*n* = 1), Thailand (*n* = 1), and the United Kingdom (*n* = 1). The oldest article identified with the search terms was from 2018.

**Table 1.** Characteristics of publications.

Category	Number (%)
Location of institutional affiliation of first author	
Australia	1 (1)
Austria	1 (1)
Bangladesh	1 (1)
Canada	9 (5)
China	2 (1.5)
Finland	1 (1)
Gabon	1 (1)
Germany	5 (3)
India	3 (1)
Iran	1 (1)
Israel	1 (1)
Luxembourg	1 (1)
Netherlands	1 (1)
Norway	1 (1)
Philippines	3 (2)
Poland	1 (1)
Spain	1 (1)
Sri Lanka	1 (1)
Sweden	1 (1)
Switzerland	2 (1.5)
Thailand	1 (1)
Türkiye	1 (1)
United Kingdom	10 (6)
United States	116 (70)
Country or region of focus	
General/global focus	139 (84)
Canada	1 (1)
Europe	3 (2)
India	1 (1)
Low- and middle-income countries	2 (2)
South Korea	1 (1)
Spain	1 (1)
Thailand	1 (1)
United Kingdom	1 (1)
United States	16 (10)
Year of publication	
2018	4 (2)
2019	4 (2)
2020	15 (9)
2021	21 (13)
2022	20 (23
2023	39 (36)
2024	59 (36)
2025	4 (2)
Primary audience of journal	
Dermatology	2 (1)
Health/healthcare in general	85 (51)
Legal	3 (2)
Medical	41 (25)
Nursing	7 (4)
Oral health	1 (<1)
Philosophy	1 (<1)
Psychology	1 (<1)
Public Health	18 (11)
Radiology	1 (<1)
Religion	1 (<1)
Sociology	1 (<1)
Technology	4 (2)
Article type	
Commentaries/editorials/opinion pieces	106 (64)
Literature reviews/theoretical frameworks	50 (30)
Scoping reviews	10 (6)
Definition of “Health Equity”	
Formal definition included within the text	22 (13)
No formal definition included	144 (87)
Focal health outcome or topic^a^	
Aging	3 (2)
Brain tumors	1 (1)
Breast cancer	1 (1)
Cancer (in general)	2 (1)
Cardiovascular conditions	9 (5)
Chronic disease	8 (5)
COVID-19	5 (3)
Diabetes mellitus	3 (2)
Disability care	1 (1)
Gynecological cancers	1 (1)
Maternal health	1 (1)
Mental health	1 (1)
Musculoskeletal conditions	1 (1)
No specific outcome/condition of focus	113 (68)
Ocular health and vision	1 (1)
Oral health	2 (1)
Prenatal and neonatal care	2 (1)
Radiology	4 (2)
Reproductive and sexual health	1 (1)
Sepsis	1 (1)
Skin cancers and dermatologic conditions	8 (5)
Surgical care	1 (1)
Women's health in general	1 (1)
Focal type of artificial intelligence addressed^a^	
AI in general	138 (83)
Natural language processing	23 (14)
Machine learning	79 (48)
Large language models	17 (10)
Generative AI	12 (7)
Predictive analytics	56 (34)
AI-powered telehealth platforms	8 (5)

^a^The total number of manuscripts in this category is greater than 166 because some articles focused on multiple outcomes or topics; the total percentage for these categories is greater than 100% because some manuscripts were multi-coded.

The disciplinary audiences of journals where manuscripts were published varied. The majority were published in general health or healthcare focused journals (51%), in addition to medicine (25%), public health (11%), nursing (4%), legal (2%), technology (2%), dermatology (1%), oral health (<1%), philosophy (<1%), psychology (<1%), radiology (<1%), religion (<1%), and sociology (<1%). Article types ranged from peer-reviewed commentaries, editorials, or opinion essays (64%), literature reviews or theoretical frameworks (30%), or scoping reviews (6%).

Only 13% (*n* = 22) of included manuscripts included an explicit definition of health equity. Of those, the most common conceptualization was modeled after the WHO definition. The majority (*n* = 15) of articles that defined health equity originated from US-based author teams with publication dates ranging from 2018 to 2024.

Almost 30% of the included articles (*n* = 48) provided a figure, framework, or chart to depict and to help facilitate equitable uses of AI in health research and practice.

Most (68%) manuscripts focused generally on health equity and AI without narrowing in on a specific health outcome or health issue. Specific health outcomes authors addressed included cardiovascular conditions (5%), chronic diseases in general (5%), dermatology and/or skin cancers (5%), COVID-19 (3%), aging (2%), diabetes mellitus (2%), radiology (2%), and others listed in Table 1.

The majority of publications characterized AI generally (83%). Specific forms of AI that were addressed included machine learning (48%), natural language processing (14%), predictive analytics (34%), large language models (10%), generative AI (7%), and AI powered telehealth platforms (5%).

### Suggestions for equitable uses of AI

The included articles’ key findings and suggestions related to the use of AI to promote health equity were both broad and specific to particular health conditions. In Table 2, we provide synthesis of the guidelines the manuscripts described related to equitable uses of AI in health/healthcare research and practice, along with exemplar articles.

**Table 2.** Selected strategies to equitably incorporate AI into healthcare systems and clinical settings, health/healthcare research, and healthcare practice.

Context	Strategies	Related articles in this review
General principles	Ethical human deliberation must accompany AI use	(Heinrichs^11^)
	Health equity must be a central goal at all stages of development for AI models	(Berdahl et al.^7^; Gurevich et al.^12^; Liao et al.^13^; Rajkomar et al.^14^)
	Diverse groups of stakeholders should engage in algorithm development and monitoring	(Hernandez-Boussard et al.^15^)
Healthcare systems and clinical settings	AI should be an epistolic tool, not the sole decision maker	(Heinrichs^11^; Hendricks-Sturrup et al.^16^)
	Implement race/ethnicity-conscious algorithms (in countries where health patterns exist across race and ethnicity)	(Cabreros et al.^17^; Istasy et al.^18^)
	Develop institutional guidelines and policies that focus on preventing harm	(Anjaria et al.^19^; Davis et al.^20^; Ferryman^21^; Thomasian et al.^6^)
	Use AI to support clinical productivity	(Johnson^22^; Johnson-Mann et al.^23^)
Health/healthcare research	Continually investigate how AI exacerbates or reduces disparities for specific populations	(Paik et al.^24^)
	Train AI on past decisions made by providers to uncover hidden inequities in provision of care	(Hendricks-Sturrup et al.^16^)
	Models must be based on representative data	(Basu et al.^25^)
Healthcare practice	Utilize AI as a support tool to enhance, not replace, human judgment in diagnosis	(Uygun et al.^26^)
	Patient–provider interaction must always accompany AI use in provision of care	(Adamson & Smith^27^; d’Elia et al.^28^; Johnson^22^; Kerr & Klonoff^29^)
	Train healthcare workers about AI and its limits	(Frehywot & Vovides^30^)

Generally, included manuscripts emphasize the critical importance of intentional human deliberation to accompany any use of AI in any healthcare context.^11^ For example, diverse groups of stakeholders and algorithm developers should coordinate before, during, and after algorithm development, as a way to reduce blind spots and mitigate unintentional algorithmic biases.^15^ Furthermore, health equity must be central at all stages of development and monitoring of AI use, not simply an afterthought.^7,12-14^

Specific to healthcare systems, manuscripts emphasized the importance of creating institutional AI guidelines and policies that focus on preventing harm. Strong regulation and built-in systems of accountability have potential to reduce unintentional bias against marginalized populations.^6,19-21^ To minimize the risk of perpetuating racialized health inequity in provision of care, authors’ emphasized the need to maintain race-conscious algorithms.^17,18^ Related to clinical settings, studies contended that AI should be an epistemic tool, not the sole decision maker.^11,16^ In an effort to combat “data chauvinism,” models in healthcare settings must be based on data that include diverse patient populations, not simply large, homogenous datasets.^25^

Regarding, health/healthcare research and practice, included manuscripts highlighted the need to continually investigate how AI can exacerbate or reduces biases/disparities for specific populations.^24^ From dermatology to chronic disease prevention, authors argued that more research is needed to assess the effects of AI use on a vast array of health outcomes across subpopulations and among specific groups.

Moreover, patient–provider interactions must *always* accompany AI use. Included manuscripts emphasized that healthcare practice cannot be reduced to quantitative algorithms that exclude the empathy, judgment, and contextual interpretation critical to a provider-patient interactions.^22,27-29^ Many authors affirmed that AI can help make timelier diagnostic decisions for patients, but it should not replace human judgment in diagnosis.^22,23^ Authors argued that AI is a beneficial support tool that can enhance, but should not replace, human judgment in provision of care.^26^ Additionally, if healthcare professionals are expected to utilize AI, then they must be trained on its basic principles and limitations.^31^

## Discussion

Our scoping review yielded 166 articles addressing equitable uses of artificial intelligence in healthcare settings, health services research, and healthcare practice. Publications included in this review emphasize that without intentional and continual human oversight, AI has potential to reliably perpetuate racialized, gendered, ableist, and other forms of bias in health research, policy, and practice. Ethical regulation and intentionality can mitigate these risks and pave the way for AI to advance health equity rather than sustain health inequities. The capabilities and uses of AI are continually expanding, making the findings of this review increasingly important. Yet, a general lack of specificity regarding what characterizes “health equity” within the context of AI, and the largely homogenous geographic and disciplinary characteristics of the included manuscripts suggest that more global perspectives and intentionality around the concept of health equity is needed.

What does health equity look like within the context of AI? Our review sought publications explicitly focused on the intersections of health equity and AI use, yet, only 13% of the identified articles provided a definition of health equity (or inequity). Over half of the articles with these definitions were published in journals representing general health/healthcare audiences. Interestingly, only 7 of these articles had primary authors from institutions outside of the United States, which suggests that there may be an over-emphasis on what equitable AI use means within US healthcare systems. Author teams based outside of the US who included a definition of health equity were most likely to apply their recommendations to chronic disease treatment and diagnosis, while US-based author teams most commonly provided general recommendations that did not apply to a specific health outcome. Of the 22 articles that included a definition of health equity, two-thirds were published since 2023, which suggests that the tendency to include a definition of health equity is more common in recent years.

Manuscripts that defined health equity most frequently utilized the WHO's definition, which articulates the centrality of justice to health equity. Heath inequities encapsulate more than health differences because the presence of an “inequity” represents present or historical injustice. As noted by Jones,^32^ health equity “requires valuing all individuals and populations equally, recognizing and rectifying historical injustices, and providing resources according to need” (p.S71). When health equity is vaguely defined, there is risk that resources will be primarily allocated to the most advantaged group, rather than the group with the greatest need.^33^ To ensure that all individuals are afforded the conditions necessary to achieve optimal health outcomes, the use of AI in healthcare research and practice should be guided by health equity principles across all stages of model design, implementation, and evaluation.

Our review highlights several areas for future research. Primarily, it underscores the need for more scholarship concerning how AI use relates to specific health outcomes and conditions. Findings suggest that specialty-specific expertise is critical for reducing the risk of exacerbating health disparities via AI usage in healthcare delivery. For example, specialists may be able to recognize how diagnostic disparities could occur because of the utilization of training data that do not include diverse and representative patient populations. For example, dermatologists likely know that historically, many clinical dermatology textbooks and public databases primarily contained images of skin lesions on lighter skin tones; if an AI tool was solely trained on traditional datasets that predominately featured patients with lighter skin, then the algorithm could struggle to detect how lesions appear on darker skin tones.^34^ While most included manuscripts focused generally on AI and health equity, specialty-specific guidance provides unique insight into *how* to avoid bias in diagnosis and healthcare delivery related to particular diseases and conditions. For example, dermatology, cardiology, and chronic disease were the most represented fields within this scoping review. To minimize the potential for harm, it may be helpful for a broader range of specialties (eg, radiologists, oncologists, gynecologists, neurologists, etc.) and health-equity researchers to partner with programmers during model design, development, implementation, and evaluation. The field needs more research investigating and highlighting how to strategically use AI to improve specific health outcomes and conditions for distinct populations and subpopulations.

Furthermore, specialty-specific insights can support creative AI-supported solutions for improved healthcare delivery. For example, integrating radiographic and biomarker information with clinical and socioeconomic data in some disciplines (eg, dentistry) makes it possible to apply advanced analytics to enable more precise diagnoses, targeted interventions, and improved health.^35^ As noted, health equity should be a central focus at *all* stages of model design, development, implementation, and evaluation.^7,12-14^ Research teams with diverse expertise—including the expertise of patients and community members—may help to mitigate harm for marginalized groups and populations with specific health conditions, while also increasing potential for AI to support the health of populations whose needs are frequently overlooked.

In addition to highlighting a gap in specialty-specific expertise, it is notable that half (51%) of the articles in this review were published in journals which represent a general health/healthcare audience, with medicine (25%) and public health (11%) facing journals being the other most prominent genres. Thus, there may be gaps in the knowledge base from less represented fields, such as nursing, oncology, or other specialties. For example, nurses are a critical factor in patients’ trust in healthcare systems; as such, nursing scientists may have unique insights regarding ethical patient education related to AI.^36^ Furthermore, allowing nurses—along with other healthcare providers—to participate in cyberethics trainings could better-equip them to utilize AI and communicate effectively with patients about AI use.^36^ In the spirit of interdisciplinary practice, more scholarship from a broader array of health disciplines and specialties could enhance providers’ and health systems’ capacity to identify and redress systemic inequities in care.

Similarly, more global perspectives on the intersections of AI and health equity are needed. None of articles’ primary authors represented an institution based in South America, and Africa and Australia were only represented by one manuscript, suggesting that the field of health equity and AI is missing the perspectives of scholars in these continents. A vast majority of manuscripts (70%) originated in the United States, suggesting that there may be overemphasis on what equitable AI implementation means within the United States's unique healthcare context. A US-centered conceptualization of health equity-driven AI implementation likely overlooks the particular needs and cultural contexts of other countries. Do practices that reduce or exacerbate health inequity in the United States have the same effect in low- or middle-income country contexts? Does AI usage differentially relate to health equity in countries with vastly different socialized or racialized disease stratification than the United States? To answer these, and other questions, future research needs to highlight the insights of global expertise. Gaining perspectives from health scholars beyond the United States would support more equitable implementation of AI into healthcare systems and healthcare practice around the world.

Furthermore, building patients’ trust in AI use requires transparency regarding how these technologies are developed and applied. At present, much of the application of AI in healthcare involves streamlining digital health platforms, such as electronic medical records and eHealth tools. Yet, AI incorporation in digital health platforms frequently translates to privatized control over patient data, potentially increasing inequity in access and understanding of these data for marginalized populations.^37^ Furthermore, these data sets rarely represent the lived experience of minoritized patient populations and frequently exclude patients who lack digital skills, which adds another level of bias if future models are trained on those data.^28,38,39^ Transparency remains a concern with AI functioning as a “black box” of information. From accountability of medical information to informed consent, patient education concerning the models utilizing their electronic health records is important to ensure that AI use is ethical and understood by all patients.^40-43^ Future research can inform how to most effectively educate patients on how their digital health data are being used within the context of AI.

Inclusive research design and methodologies could also help ensure that AI is applied in ways that promote health for *all* populations. AI is not neutral, and it is paramount to train AI on representative datasets. Large, homogenous datasets used by health systems frequently lack subgroups, which can translate into unequal clinical outcomes across distinct groups of people.^40-43^ This includes, undertreatment, delayed care, and even misdiagnoses for minoritized populations.^25,44,45^ Health services researchers and leaders of health systems must be aware of and understand potential biases in data sources before broadly applying them. This could help prevent the development of spurious or biased model relationships, which perpetuate inequity in healthcare services and health outcomes.^46-48^ To ensure that data are representative, decision-makers must understand which social or demographic categories are most important within their respective social or geographic context. For example, in the context of the United States, race-conscious and gender-conscious algorithms are encouraged because there are historical and reliable patterns in health differences, disparities, and broader inequities across race and gender. Expanding AI education and training for health researchers and health system leaders could better equip them to recognize and mitigate how bias is embedded into the training data.^46^

While AI continues to advance rapidly across clinical and operational domains, robust regulatory and institutional oversight—explicitly grounded in an equity framework—is essential to ensure its ethical, trustworthy, and just application. The establishment of standards for fairness, transparency, accountability, and data privacy is critical for mitigating bias, reducing potential harms, and safeguarding patient rights. Such governance mechanisms are also foundational to building public trust through rigorous validation processes, continuous oversight, and clear communication regarding the role of AI in decision-making.

Additional research is needed to determine if and how the health equity-based recommendations highlighted in this review are being implemented within healthcare systems in disparate regional and international contexts. In 2024 the European Union passed the European Artificial Intelligence Act (AI Act), a comprehensive set of regulations stating that AI-based software used for medical purposes must include “risk-mitigation systems, high-quality data sets, clear user information, and human oversight.”^49^ While the European AI-Act has been criticized for its lack of attention to the importance of building trustworthiness of AI within healthcare systems,^50^ it explicitly states that AI should be regulated in ways that ensure health, safety, and fundamental rights.^49^ This goes beyond the language currently used in many of the current United States-based regulatory frameworks, which have prioritized the deregulation of AI since early 2025.^51^ Future investigations could provide insights into the ways in which regulations and deregulations are affecting population health equity in similar or different ways around the world.

We encourage grant funders and academic journals to require researchers using AI to explicitly include definitions of equity and reporting on subgroups and marginalized populations. Similarly, healthcare systems could adopt and implement explicit, equity-focused governance of AI so that there is continual oversight and safeguards in place to reduce risks of harm. Furthermore, regulatory approaches informed by interdisciplinary collaboration can advance health equity by promoting the use of representative data, systematically measuring and monitoring disparities, and ensuring equitable access to personalized, AI-enabled innovations across diverse populations. We contend that national regulatory agencies should incorporate frameworks based on the WHO's definition of healthy equity into their evaluations. Deliberate and sustained attention to these safeguards will be necessary to support responsible innovation that aligns with societal values and contributes to improved population health outcomes.

### Limitations

As with any scoping review, a limitation of our findings is that other manuscripts on this topic have been published since the time we conducted our searches. However, many of these publications affirm our findings and emphasize the need for continual discourse on AI and health equity. Another limitation is that all included manuscripts were published in English, meaning that there are likely health scholars’ perspectives that are not represented. Because we included scoping reviews in our analysis, it is also possible that the scoping reviews already capture the content from the literature reviews, theoretical frameworks, opinion pieces, editorials, and commentaries we included in this review. As such, there may be an over-emphasis on certain recommendations from manuscripts that were included in our review and earlier scoping reviews.

Given the broad nature of our research aims, it was beyond the scope of this review to summarize the exemplary discipline-specific (eg, aging, women's health) and health outcome-specific (eg, cancer, chronic disease) recommendations represented in some of the included publications. For that reason, we encourage readers to utilize Appendix 2 to find more information on particular topics of interest. Because our review focused on collecting and synthesizing guidelines and suggestions rather than assessing the outcomes of research studies and randomized controlled trials, we were not able to identify if the suggestions from the included manuscripts are linked to meaningful or significant reductions in health inequity related to specific health outcomes. Future research could explore this issue.

## Conclusion

Despite these limitations, our review offers a unique synthesis of recommendations for equitable integration of AI into healthcare settings, health research, and healthcare practice. Included manuscripts emphasize that ethical human oversight must accompany AI use. Furthermore, engaging discipline-specific expertise and diverse groups of stakeholders during model development, implementation, and evaluation would reduce model bias and improve the quality of care provided. A broader depth of research highlighting global perspectives would enhance our ability to use AI in ways that reduce health disparities and inequities for specific populations. Moreover, patient and provider education on the capabilities of AI are also critical for building trust and ensuring equitable delivery of care. By prioritizing transparency, inclusion, and education, there is potential to meaningfully regulate AI in healthcare to redress systemic inequities in care and advance health and well-being in populations throughout the world.

## Supplementary Material

qxag236_Supplementary_Data
