# Supplementary material for: Recommended guidelines concerning artificial intelligence and health equity: a scoping review
Source: Health Aff Sch. 2026 Oct 8;4(10):qxag236. doi: 10.1093/haschl/qxag236 (PMC13648153; doi:10.1093/haschl/qxag236)
Supplement: Supplementary file 1 [file qxag236_supplementary_data.zip › APPENDIX 1 - 8.13.2026.docx]

**APPENDIX 1**

| **PubMed (period searched 1/1/2014 to 1/22/2025, NIH.gov) Search Terms** | | |
| --- | --- | --- |
| 1 | (((("inequalit*"[Title] OR "equalit*"[Title] OR "inequit*"[Title] OR "equit*"[Title] OR "justice"[Title] OR "disparit*"[Title] OR "differenc*"[Title]) AND ("health"[Title] OR "healthcare"[Title])) OR ("health equity"[MeSH Terms] OR "healthcare disparities"[MeSH Terms])) AND ("artificial intelligence"[Title/Abstract] OR "ai"[Title/Abstract] OR "natural language processing"[Title/Abstract] OR "machine learning"[Title/Abstract] OR "large language models"[Title/Abstract] OR "deep learning"[Title/Abstract] OR "artificial intelligence"[MeSH Terms] OR "machine learning"[MeSH Terms] OR "natural language processing"[MeSH Terms])) AND (("2014/01/01"[Date - Publication] : "3000"[Date - Publication])) | 444 |
