# Supplementary material for: Recommended guidelines concerning artificial intelligence and health equity: a scoping review
Source: Health Aff Sch. 2026 Oct 8;4(10):qxag236. doi: 10.1093/haschl/qxag236 (PMC13648153; doi:10.1093/haschl/qxag236)
Supplement: Supplementary file 1 [file qxag236_supplementary_data.zip › APPENDIX 2 - 8.13.2026.docx]

**APPENDIX 2**

**Manuscripts Included in the Review:**

Abdel Magid HS, Desjardins MR, Hu Y. Opportunities and shortcomings of AI for spatial epidemiology and health disparities research on aging and the life course. *Health Place*. 2024;89:103323. doi:[10.1016/j.healthplace.2024.103323](https://doi.org/10.1016/j.healthplace.2024.103323)

Abràmoff MD, Tarver ME, Loyo-Berrios N, et al. Considerations for addressing bias in artificial intelligence for health equity. *NPJ Digit Med*. 2023;6(1):170. doi:[10.1038/s41746-023-00913-9](https://doi.org/10.1038/s41746-023-00913-9)

Adamson AS, Smith A. Machine Learning and Health Care Disparities in Dermatology. *JAMA Dermatol*. 2018;154(11):1247-1248. doi:[10.1001/jamadermatol.2018.2348](https://doi.org/10.1001/jamadermatol.2018.2348)

Adedinsewo D. Advancing Cardiovascular Health Equity With Artificial Intelligence: A Collective Ethical Responsibility. *Circulation*. 2024;150(3):174-176. doi:[10.1161/CIRCULATIONAHA.124.068113](https://doi.org/10.1161/CIRCULATIONAHA.124.068113)

Afroze L, Rahman MS. Utilization of artificial intelligence to mitigate health inequalities in gynecological cancer care. *Int J Gynecol Cancer*. 2024;34(10):1657-1658. doi:[10.1136/ijgc-2024-005788](https://doi.org/10.1136/ijgc-2024-005788)

Agarwal R, Gao G. Toward an “Equitable” Assimilation of Artificial Intelligence and Machine Learning into Our Health Care System. *N C Med J*. 2024;85(4):246-250. doi:[10.18043/001c.120565](https://doi.org/10.18043/001c.120565)

Agarwal R, Bjarnadottir M, Rhue L, et al. Addressing algorithmic bias and the perpetuation of health inequities: An AI bias aware framework. *Health Policy and Technology*. 2023;12(1). doi:[10.1016/j.hlpt.2022.100702](https://doi.org/10.1016/j.hlpt.2022.100702)

Agarwal R, Bjarnadottir M, Clark J, Rhue L, Gao GG. Data Science for Health Equity: Perils and Promise of AI/ML in Healthcare. *ML in Healthcare (April 24, 2023)*. Published online 2023.

Ali MR, Lawson CA, Wood AM, Khunti K. Addressing ethnic and global health inequalities in the era of artificial intelligence healthcare models: a call for responsible implementation. *J R Soc Med*. 2023;116(8):260-262. doi:[10.1177/01410768231187734](https://doi.org/10.1177/01410768231187734)

Amponsah D, Thamman R, Brandt E, James C, Spector-Bagdady K, Yong C. Artificial Intelligence to Promote Racial and Ethnic Cardiovascular Health Equity. *CURRENT CARDIOVASCULAR RISK REPORTS*. 2024;18(11):153-162. doi:[10.1007/s12170-024-00745-6](https://doi.org/10.1007/s12170-024-00745-6)

Amugongo L, Bidwell N, Association of Computing Machinery, Corrigan C. Invigorating Ubuntu Ethics in AI for healthcare: Enabling equitable care. In: 2023:583-592. doi:[10.1145/3593013.3594024](https://doi.org/10.1145/3593013.3594024)

Anjaria P, Asediya V, Bhavsar P, Pathak A, Desai D, Patil V. Artificial Intelligence in Public Health: Revolutionizing Epidemiological Surveillance for Pandemic Preparedness and Equitable Vaccine Access. *Vaccines (Basel)*. 2023;11(7). doi:[10.3390/vaccines11071154](https://doi.org/10.3390/vaccines11071154)

Asabor EN, Aneni K, Weerakoon S, Opara I. Applying a community‐engaged participatory machine learning model. *American Journal of Community Psychology*. 2024;74(3/4):262-268.

Azizi Z, Vedelli JKH, Anand KJS. Informative Artifacts in AI-Assisted Care. *New England Journal of Medicine*. 2023;389(22):2113-2113.

Bagheri AB, Rouzi MD, Koohbanani NA, et al. Potential applications of artificial intelligence and machine learning on diagnosis, treatment, and outcome prediction to address health care disparities of chronic limb-threatening ischemia. *Semin Vasc Surg*. 2023;36(3):454-459. doi:[10.1053/j.semvascsurg.2023.06.003](https://doi.org/10.1053/j.semvascsurg.2023.06.003)

Bakdash L, Abid A, Gourisankar A, Henry TL. Chatting Beyond ChatGPT: Advancing Equity Through AI-Driven Language Interpretation. *J Gen Intern Med*. 2024;39(3):492-495. doi:[10.1007/s11606-023-08497-6](https://doi.org/10.1007/s11606-023-08497-6)

Balagopalan A, Baldini I, Celi LA, et al. Machine learning for healthcare that matters: Reorienting from technical novelty to equitable impact. *PLOS Digit Health*. 2024;3(4):e0000474. doi:[10.1371/journal.pdig.0000474](https://doi.org/10.1371/journal.pdig.0000474)

Barton M, Hamza M, Guevel B. Racial Equity in Healthcare Machine Learning: Illustrating Bias in Models With Minimal Bias Mitigation. *Cureus*. 2023;15(2):e35037. doi:[10.7759/cureus.35037](https://doi.org/10.7759/cureus.35037)

Basu S, Faghmous JH, Doupe P. Machine Learning Methods for Precision Medicine Research Designed to Reduce Health Disparities: A Structured Tutorial. *Ethn Dis*. 2020;30(Suppl 1):217-228. doi:[10.18865/ed.30.S1.217](https://doi.org/10.18865/ed.30.S1.217)

Baumgartner R, Arora P, Bath C, et al. Fair and equitable AI in biomedical research and healthcare: Social science perspectives. *Artif Intell Med*. 2023;144:102658. doi:[10.1016/j.artmed.2023.102658](https://doi.org/10.1016/j.artmed.2023.102658)

Bayne J, Garry J, Albert MA. Brief Review: Racial and Ethnic Disparities in Cardiovascular Care with a Focus on Congenital Heart Disease and Precision Medicine. *Curr Atheroscler Rep*. 2023;25(5):189-195. doi:[10.1007/s11883-023-01093-3](https://doi.org/10.1007/s11883-023-01093-3)

Bechtel MA. Artificial Intelligence: An Emerging Challenge for State Medical Boards. *Journal of Medical Regulation*. 2024;110(1):6-6.

Berdahl CT, Baker L, Mann S, Osoba O, Girosi F. Strategies to Improve the Impact of Artificial Intelligence on Health Equity: Scoping Review. *JMIR AI*. 2023;2:e42936. doi:[10.2196/42936](https://doi.org/10.2196/42936)

Bobrowski D, Joshi H. Unmasking A.I.’s bias in healthcare: The need for diverse data. *University of Toronto Medical Journal*. 2019;96(1):48-50.

Bowser DM, Maurico K, Ruscitti BA, Crown WH. American clusters: using machine learning to understand health and health care disparities in the United States. *Health Aff Sch*. 2024;2(3):qxae017. doi:[10.1093/haschl/qxae017](https://doi.org/10.1093/haschl/qxae017)

Bridges KM. RACE IN THE MACHINE: RACIAL DISPARITIES IN HEALTH AND MEDICAL AI. *Virginia Law Review*. 2024;110(2):243-339.

Cabreros I, Agniel D, Martino SC, Damberg CL, Elliott MN. Predicting Race And Ethnicity To Ensure Equitable Algorithms For Health Care Decision Making. *Health Aff (Millwood)*. 2022;41(8):1153-1159. doi:[10.1377/hlthaff.2022.00095](https://doi.org/10.1377/hlthaff.2022.00095)

Campbell JP, Mathenge C, Cherwek H, et al. Artificial Intelligence to Reduce Ocular Health Disparities: Moving From Concept to Implementation. *Transl Vis Sci Technol*. 2021;10(3):19. doi:[10.1167/tvst.10.3.19](https://doi.org/10.1167/tvst.10.3.19)

Cary MP Jr, Bessias S, McCall J, et al. Empowering nurses to champion Health equity & BE FAIR: Bias elimination for fair and responsible AI in healthcare. *J Nurs Scholarsh*. Published online 2024. doi:[10.1111/jnu.13007](https://doi.org/10.1111/jnu.13007)

Cary MP Jr, De Gagne JC, Kauschinger ED, Carter BM. Advancing Health Equity Through Artificial Intelligence: An Educational Framework for Preparing Nurses in Clinical Practice and Research. *Creat Nurs*. 2024;30(2):154-164. doi:[10.1177/10784535241249193](https://doi.org/10.1177/10784535241249193)

Celi LA, Cellini J, Charpignon ML, et al. Sources of bias in artificial intelligence that perpetuate healthcare disparities-A global review. *PLOS Digit Health*. 2022;1(3):e0000022. doi:[10.1371/journal.pdig.0000022](https://doi.org/10.1371/journal.pdig.0000022)

Chan SCC, Neves AL, Majeed A, Faisal A. Bridging the equity gap towards inclusive artificial intelligence in healthcare diagnostics. *BMJ*. 2024;384:q490. doi:[10.1136/bmj.q490](https://doi.org/10.1136/bmj.q490)

Char DS, Shah NH, Magnus D. Implementing Machine Learning in Health Care - Addressing Ethical Challenges. *N Engl J Med*. 2018;378(11):981-983. doi:[10.1056/NEJMp1714229](https://doi.org/10.1056/NEJMp1714229)

Chase AC. Ethics of AI: Perpetuating racial inequalities in healthcare delivery and patient outcomes. *Voices in Bioethics*. 2020;6. <https://journals.library.columbia.edu/index.php/bioethics/article/view/5890>

Chen IY, Joshi S, Ghassemi M. Treating health disparities with artificial intelligence. *Nat Med*. 2020;26(1):16-17. doi:[10.1038/s41591-019-0649-2](https://doi.org/10.1038/s41591-019-0649-2)

Chen Y, Clayton EW, Novak LL, Anders S, Malin B. Human-Centered Design to Address Biases in Artificial Intelligence. *J Med Internet Res*. 2023;25:e43251. doi:[10.2196/43251](https://doi.org/10.2196/43251)

Cheraghlou S. In defence of the machines: How artificial intelligence may help to improve dermatologic outcomes and diminish barriers and disparities in care. *Australas J Dermatol*. 2023;64(1):e106-e107. doi:[10.1111/ajd.13956](https://doi.org/10.1111/ajd.13956)

Cirillo D, Catuara-Solarz S, Morey C, et al. Sex and gender differences and biases in artificial intelligence for biomedicine and healthcare. *NPJ Digit Med*. 2020;3:81. doi:[10.1038/s41746-020-0288-5](https://doi.org/10.1038/s41746-020-0288-5)

Clark CR, Wilkins CH, Rodriguez JA, et al. Health Care Equity in the Use of Advanced Analytics and Artificial Intelligence Technologies in Primary Care. *J Gen Intern Med*. 2021;36(10):3188-3193. doi:[10.1007/s11606-021-06846-x](https://doi.org/10.1007/s11606-021-06846-x)

Crawford AD, Slavin R, Tabar M, et al. Methodological approaches in developing and implementing digital health interventions amongst underserved women. *Public Health Nursing*. 2024;41(6):1612-1621.

d’Elia A, Gabbay M, Rodgers S, et al. Artificial intelligence and health inequities in primary care: a systematic scoping review and framework. *Fam Med Community Health*. 2022;10(Suppl 1). doi:[10.1136/fmch-2022-001670](https://doi.org/10.1136/fmch-2022-001670)

da Silva JB Jr, Lima NT, Haddad AE, et al. From national and regional commitments to global impact: artificial intelligence for equitable public health at the G20. *Rev Panam Salud Publica*. 2024;48:e73. doi:[10.26633/RPSP.2024.73](https://doi.org/10.26633/RPSP.2024.73)

Dankwa-Mullan I. Health Equity and Ethical Considerations in Using Artificial Intelligence in Public Health and Medicine. *Prev Chronic Dis*. 2024;21:E64. doi:[10.5888/pcd21.240245](https://doi.org/10.5888/pcd21.240245)

Dankwa-Mullan I, Ndoh K, Akogo D, Rocha HAL, Juaçaba SF. Artificial Intelligence and Cancer Health Equity: Bridging the Divide or Widening the Gap. *Curr Oncol Rep*. Published online 2025. doi:[10.1007/s11912-024-01627-1](https://doi.org/10.1007/s11912-024-01627-1)

Dankwa-Mullan I, Scheufele E, Matheny M, et al. A Proposed Framework on Integrating Health Equity and Racial Justice into the Artificial Intelligence Development Lifecycle. *JOURNAL OF HEALTH CARE FOR THE POOR AND UNDERSERVED*. 2021;32(2):300-317. doi:[10.1353/hpu.2021.0065](https://doi.org/10.1353/hpu.2021.0065)

Davis MA, Lim N, Jordan J, Yee J, Gichoya JW, Lee R. Imaging Artificial Intelligence: A Framework for Radiologists to Address Health Equity, From the AJR Special Series on DEI. *AJR Am J Roentgenol*. 2023;221(3):302-308. doi:[10.2214/AJR.22.28802](https://doi.org/10.2214/AJR.22.28802)

Delgado J, de Manuel A, Parra I, et al. Bias in algorithms of AI systems developed for COVID-19: A scoping review. *Journal of Bioethical Inquiry*. 2022;19(3):407-419.

Demuro P, Petersen C, Turner P. Health “Big Data” Value, Benefit, and Control: The Patient eHealth Equity Gap. *Stud Health Technol Inform*. 2020;270:1123-1127. doi:[10.3233/SHTI200337](https://doi.org/10.3233/SHTI200337)

Deo RC. Artificial Intelligence and Health Equity. *DEPUTY EDITORS*. Published online 2024.

Dolatkhah Laein G. Global perspectives on governing healthcare AI: prioritising safety, equity and collaboration. *BMJ Lead*. Published online 2024. doi:[10.1136/leader-2023-000904](https://doi.org/10.1136/leader-2023-000904)

Dychiao RG, Nazer L, Mlombwa D, Celi LA. Artificial intelligence and global health equity. *BMJ*. 2024;387:q2194. doi:[10.1136/bmj.q2194](https://doi.org/10.1136/bmj.q2194)

Editorial Board of Nature Machine Learning. Striving for health equity with machine learning. *NATURE MACHINE INTELLIGENCE*. 2021;3(8):653-653. doi:[10.1038/s42256-021-00385-0](https://doi.org/10.1038/s42256-021-00385-0)

Elani HW, Giannobile WV. Harnessing Artificial Intelligence to Address Oral Health Disparities. *JAMA Health Forum*. 2024;5(4):e240642. doi:[10.1001/jamahealthforum.2024.0642](https://doi.org/10.1001/jamahealthforum.2024.0642)

Ellen JG, Matos J, Viola M, et al. Participant flow diagrams for health equity in AI. *J Biomed Inform*. 2024;152:104631. doi:[10.1016/j.jbi.2024.104631](https://doi.org/10.1016/j.jbi.2024.104631)

Embi PJ. Algorithmovigilance-Advancing Methods to Analyze and Monitor Artificial Intelligence-Driven Health Care for Effectiveness and Equity. *JAMA Netw Open*. 2021;4(4):e214622. doi:[10.1001/jamanetworkopen.2021.4622](https://doi.org/10.1001/jamanetworkopen.2021.4622)

Feehan M, Owen LA, McKinnon IM, DeAngelis MM. Artificial Intelligence, Heuristic Biases, and the Optimization of Health Outcomes: Cautionary Optimism. *Journal of Clinical Medicine*. 2021;10(22):5284-5284.

Ferryman K. Addressing health disparities in the Food and Drug Administration’s artificial intelligence and machine learning regulatory framework. *J Am Med Inform Assoc*. 2020;27(12):2016-2019. doi:[10.1093/jamia/ocaa133](https://doi.org/10.1093/jamia/ocaa133)

Ferryman K, Macintosh M, Ghassemi M. Informative Artifacts in AI-Assisted Care. *New England Journal of Medicine*. 2023;389(22):2114-2115.

Ferryman K, Mackintosh M, Ghassemi M. Considering Biased Data as Informative Artifacts in AI-Assisted Health Care. *New England Journal of Medicine*. 2023;389(9):833-838.

Franklin G, Stephens R, Piracha M, et al. The Sociodemographic Biases in Machine Learning Algorithms: A Biomedical Informatics Perspective. *Life (2075-1729)*. 2024;14(6):652.

Frehywot S, Vovides Y. An equitable and sustainable community of practice framework to address the use of artificial intelligence for global health workforce training. *Hum Resour Health*. 2023;21(1):45. doi:[10.1186/s12960-023-00833-5](https://doi.org/10.1186/s12960-023-00833-5)

Frennert S. Gender blindness: On health and welfare technology, AI and gender equality in community care. *Nurs Inq*. 2021;28(4):e12419. doi:[10.1111/nin.12419](https://doi.org/10.1111/nin.12419)

Gerybaite A, Palmieri S, Vigna F. Equality in Healthcare AI: Did Anyone Mention Data Quality? *BIOLAW JOURNAL-RIVISTA DI BIODIRITTO*. 2022;(4):385-409.

Goldust M. Utilizing AI to address skin disorders and healthcare disparities among undocumented immigrants. *Int J Dermatol*. 2024;63(7):e123. doi:[10.1111/ijd.17208](https://doi.org/10.1111/ijd.17208)

Goodman SN, Goel S, Cullen MR. Machine Learning, Health Disparities, and Causal Reasoning. *Ann Intern Med*. 2018;169(12):883-884. doi:[10.7326/M18-3297](https://doi.org/10.7326/M18-3297)

Gozum IEA. How can the principle of justice as fairness help address the digital divide in AI and healthcare? *J Public Health (Oxf)*. Published online 2024. doi:[10.1093/pubmed/fdae163](https://doi.org/10.1093/pubmed/fdae163)

Gozum IEA, Flake CCD. Integrating Catholic Social Teaching with AI Ethics to Address Inequity in AI Healthcare. *J Relig Health*. 2024;63(6):4323-4341. doi:[10.1007/s10943-024-02140-2](https://doi.org/10.1007/s10943-024-02140-2)

Green BL, Murphy A, Robinson E. Accelerating health disparities research with artificial intelligence. *Front Digit Health*. 2024;6:1330160. doi:[10.3389/fdgth.2024.1330160](https://doi.org/10.3389/fdgth.2024.1330160)

Grzybowski A, Jin K, Wu H. Challenges of artificial intelligence in medicine and dermatology. *Clin Dermatol*. 2024;42(3):210-215. doi:[10.1016/j.clindermatol.2023.12.013](https://doi.org/10.1016/j.clindermatol.2023.12.013)

Gurevich E, El Hassan B, El Morr C. Equity within AI systems: What can health leaders expect? *Healthc Manage Forum*. 2023;36(2):119-124. doi:[10.1177/08404704221125368](https://doi.org/10.1177/08404704221125368)

Gwillim EC, Azzawi S, Aigen AR. Underserved populations and health equity in dermatology: Digital medicine and the role of artificial intelligence. *Clin Dermatol*. 2024;42(5):498-506. doi:[10.1016/j.clindermatol.2024.06.021](https://doi.org/10.1016/j.clindermatol.2024.06.021)

Hamam H. Guest Editorial Achieving Health Equity Through AI for Diagnosis and Treatment and Patient Monitoring. *IEEE JOURNAL OF BIOMEDICAL AND HEALTH INFORMATICS*. 2024;28(2):702-706. doi:[10.1109/JBHI.2023.3348249](https://doi.org/10.1109/JBHI.2023.3348249)

Hartzler AL, Xie SJ, Wedgeworth P, et al. Integrating patient voices into the extraction of social determinants of health from clinical notes: ethical considerations and recommendations. *J Am Med Inform Assoc*. 2023;30(8):1456-1462. doi:[10.1093/jamia/ocad043](https://doi.org/10.1093/jamia/ocad043)

Heinrichs JH. Hammer or Measuring Tape? Artificial Intelligence and Justice in Healthcare. *Cambridge Quarterly of Healthcare Ethics*. 2024;33(3):1-12.

Hendricks-Sturrup R, Simmons M, Anders S, et al. Developing Ethics and Equity Principles, Terms, and Engagement Tools to Advance Health Equity and Researcher Diversity in AI and Machine Learning: Modified Delphi Approach. *JMIR AI*. 2023;2:e52888. doi:[10.2196/52888](https://doi.org/10.2196/52888)

Hernandez-Boussard T, Siddique SM, Bierman AS, Hightower M, Burstin H. Promoting Equity In Clinical Decision Making: Dismantling Race-Based Medicine. *Health Aff (Millwood)*. 2023;42(10):1369-1373. doi:[10.1377/hlthaff.2023.00545](https://doi.org/10.1377/hlthaff.2023.00545)

Holzmeyer C. Beyond “AI for Social Good” (AI4SG): social transformations—not tech-fixes—for health equity. *Interdisciplinary Science Reviews*. 2021;46(1/2):94-125.

Homanen R, McBride N, Hudson N. Artificial intelligence and assisted reproductive technology: Applying a reproductive justice lens. *European Journal of Women’s Studies*. 2024;31(2):262-276.

Hosny A, Aerts HJWL. Artificial intelligence for global health: Socially responsible technologies promise to help address health care inequalities. *Science*. 2019;366(6468):955-956.

Ibrahim SA, Pronovost PJ. Diagnostic Errors, Health Disparities, and Artificial Intelligence: A Combination for Health or Harm? *JAMA Health Forum*. 2021;2(9):e212430. doi:[10.1001/jamahealthforum.2021.2430](https://doi.org/10.1001/jamahealthforum.2021.2430)

Istasy P, Lee W, Iansavitchene A, et al. The Impact of Artificial Intelligence on Health Equity in Oncology: A Scoping Review. *BLOOD*. 2021;138. doi:[10.1182/blood-2021-149264](https://doi.org/10.1182/blood-2021-149264)

Johnson AE, Brewer LC, Echols MR, Mazimba S, Shah RU, Breathett K. Utilizing Artificial Intelligence to Enhance Health Equity Among Patients with Heart Failure. *Heart Fail Clin*. 2022;18(2):259-273. doi:[10.1016/j.hfc.2021.11.001](https://doi.org/10.1016/j.hfc.2021.11.001)

Johnson SLJ. AI, Machine Learning, and Ethics in Health Care. *J Leg Med*. 2019;39(4):427-441. doi:[10.1080/01947648.2019.1690604](https://doi.org/10.1080/01947648.2019.1690604)

Johnson-Mann CN, Loftus TJ, Bihorac A. Equity and Artificial Intelligence in Surgical Care. *JAMA Surg*. 2021;156(6):509-510. doi:[10.1001/jamasurg.2020.7208](https://doi.org/10.1001/jamasurg.2020.7208)

Joshi A. Big data and AI for gender equality in health: bias is a big challenge. *Front Big Data*. 2024;7:1436019. doi:[10.3389/fdata.2024.1436019](https://doi.org/10.3389/fdata.2024.1436019)

Kalusivalingam AK, Sharma A, Patel N, Singh V. Leveraging Federated Learning and Explainable AI for Advancing Health Equity: A Comprehensive Approach to Reducing Disparities in Healthcare Access and Outcomes. *International Journal of AI and ML*. 2021;2(3).

Kamyabi A, Iyamu I, Saini M, May C, McKee G, Choi A. Advocating for population health: The role of public health practitioners in the age of artificial intelligence. *Can J Public Health*. 2024;115(3):473-476. doi:[10.17269/s41997-024-00881-x](https://doi.org/10.17269/s41997-024-00881-x)

Kapoor N, Sanjana SN, Davalagi SB, Balu PS, Sethia S. AI Horizons in Indian Healthcare: A Vision for Transformation and Equity. *Indian Journal of Community Medicine*. 49:S210-S216.

Kempt H, Freyer N, Nagel SK. Justice and the Normative Standards of Explainability in Healthcare. *Philosophy & Technology*. 2022;35(4):1-22.

Kerr D, Klonoff DC. Digital Diabetes Data and Artificial Intelligence: A Time for Humility Not Hubris. *J Diabetes Sci Technol*. 2019;13(1):123-127. doi:[10.1177/1932296818796508](https://doi.org/10.1177/1932296818796508)

Khera R, Butte AJ, Berkwits M, et al. AI in Medicine-JAMA’s Focus on Clinical Outcomes, Patient-Centered Care, Quality, and Equity. *JAMA*. 2023;330(9):818-820. doi:[10.1001/jama.2023.15481](https://doi.org/10.1001/jama.2023.15481)

Khoury ZH, Ferguson A, Price JB, Sultan AS, Wang R. Responsible artificial intelligence for addressing equity in oral healthcare. *Front Oral Health*. 2024;5:1408867. doi:[10.3389/froh.2024.1408867](https://doi.org/10.3389/froh.2024.1408867)

Kim JY, Hasan A, Kellogg KC, et al. Development and preliminary testing of Health Equity Across the AI Lifecycle (HEAAL): A framework for healthcare delivery organizations to mitigate the risk of AI solutions worsening health inequities. *PLOS Digit Health*. 2024;3(5):e0000390. doi:[10.1371/journal.pdig.0000390](https://doi.org/10.1371/journal.pdig.0000390)

Kocher MR, Lee CI. Preventing Artificial Intelligence in Medical Imaging From Perpetuating Health Care Biases and Disparities. *J Am Coll Radiol*. 2022;19(12):1345-1346. doi:[10.1016/j.jacr.2022.07.021](https://doi.org/10.1016/j.jacr.2022.07.021)

Koranteng E, Rao A, Flores E, et al. Empathy and Equity: Key Considerations for Large Language Model Adoption in Health Care. *JMIR Med Educ*. 2023;9:e51199. doi:[10.2196/51199](https://doi.org/10.2196/51199)

Lawson McLean A. Towards Precision Medicine in Spinal Surgery: Leveraging AI Technologies. *Ann Biomed Eng*. 2024;52(4):735-737. doi:[10.1007/s10439-023-03315-w](https://doi.org/10.1007/s10439-023-03315-w)

Lee CI, Elmore JG. Cancer Risk Prediction Paradigm Shift: Using Artificial Intelligence to Improve Performance and Health Equity. *J Natl Cancer Inst*. 2022;114(10):1317-1319. doi:[10.1093/jnci/djac143](https://doi.org/10.1093/jnci/djac143)

Lee EWJ, Viswanath K. Big Data in Context: Addressing the Twin Perils of Data Absenteeism and Chauvinism in the Context of Health Disparities Research. *J Med Internet Res*. 2020;22(1):e16377. doi:[10.2196/16377](https://doi.org/10.2196/16377)

Leslie D, Mazumder A, Peppin A, Wolters MK, Hagerty A. Does “AI” stand for augmenting inequality in the era of covid-19 healthcare? *BMJ*. 2021;372:n304. doi:[10.1136/bmj.n304](https://doi.org/10.1136/bmj.n304)

Li H, Moon JT, Shankar V, Newsome J, Gichoya J, Bercu Z. Health inequities, bias, and artificial intelligence. *Tech Vasc Interv Radiol*. 2024;27(3):100990. doi:[10.1016/j.tvir.2024.100990](https://doi.org/10.1016/j.tvir.2024.100990)

Liao F, Adelaine S, Afshar M, Patterson BW. Governance of Clinical AI applications to facilitate safe and equitable deployment in a large health system: Key elements and early successes. *Front Digit Health*. 2022;4:931439. doi:[10.3389/fdgth.2022.931439](https://doi.org/10.3389/fdgth.2022.931439)

Liebrenz M, Bhugra D, Alibudbud R, Ventriglio A, Smith A. AI in health care and the fragile pursuit of equity and social justice. *Lancet*. 2024;404(10455):843. doi:[10.1016/S0140-6736(24)01604-0](https://doi.org/10.1016/S0140-6736(24)01604-0)

Makhni S, Chin MH, Fahrenbach J, Rojas JC. Equity Challenges for Artificial Intelligence Algorithms in Health Care. *Chest*. 2022;161(5):1343-1346. doi:[10.1016/j.chest.2022.01.009](https://doi.org/10.1016/j.chest.2022.01.009)

Manríquez Roa T, Christen M, Reis A, Biller-Andorno N. The pursuit of health equity in the era of artificial intelligence. *Swiss Med Wkly*. 2023;153:40062. doi:[10.57187/smw.2023.40062](https://doi.org/10.57187/smw.2023.40062)

Maw AM, Trinkley KE, Glasgow RE. The Role of Pragmatic Implementation Science Methods in Achieving Equitable and Effective Use of Artificial Intelligence in Healthcare. *J Gen Intern Med*. 2024;39(7):1242-1244. doi:[10.1007/s11606-023-08580-y](https://doi.org/10.1007/s11606-023-08580-y)

McAdams RM, Green TL. Equitable Artificial Intelligence in Obstetrics, Maternal–Fetal Medicine, and Neonatology. *Obstetrics & Gynecology*. 2024;143(5):627-632.

McCradden MD, Joshi S, Anderson JA, Mazwi M, Goldenberg A, Zlotnik Shaul R. Patient safety and quality improvement: Ethical principles for a regulatory approach to bias in healthcare machine learning. *J Am Med Inform Assoc*. 2020;27(12):2024-2027. doi:[10.1093/jamia/ocaa085](https://doi.org/10.1093/jamia/ocaa085)

McCradden MD, Joshi S, Mazwi M, Anderson JA. Ethical limitations of algorithmic fairness solutions in health care machine learning. *Lancet Digit Health*. 2020;2(5):e221-e223. doi:[10.1016/S2589-7500(20)30065-0](https://doi.org/10.1016/S2589-7500(20)30065-0)

Mehari M, Sibih Y, Dada A, et al. Enhancing neuro-oncology care through equity-driven applications of artificial intelligence. *Neuro Oncol*. 2024;26(11):1951-1963. doi:[10.1093/neuonc/noae127](https://doi.org/10.1093/neuonc/noae127)

Mitchell WG, Dee EC, Celi LA. Generalisability through local validation: overcoming barriers due to data disparity in healthcare. *BMC Ophthalmol*. 2021;21(1):228. doi:[10.1186/s12886-021-01992-6](https://doi.org/10.1186/s12886-021-01992-6)

Molli VLP. Enhancing Healthcare Equity through AI-Powered Decision Support Systems: Addressing Disparities in Access and Treatment Outcomes. *International Journal of Sustainable Development Through AI, ML and IoT*. 2024;3(1):1-12.

Moore CM. The challenges of health inequities and AI. *Intelligence-Based Medicine*. 2022;6:100067.

Murphy A, Bowen K, Naqa IME, Yoga B, Green BL. Bridging Health Disparities in the Data-Driven World of Artificial Intelligence: A Narrative Review. *J Racial Ethn Health Disparities*. Published online 2024. doi:[10.1007/s40615-024-02057-2](https://doi.org/10.1007/s40615-024-02057-2)

Newman-Griffis DR, Hurwitz MB, McKernan GP, Houtrow AJ, Dicianno BE. A roadmap to reduce information inequities in disability with digital health and natural language processing. *PLOS Digit Health*. 2022;1(11):e0000135. doi:[10.1371/journal.pdig.0000135](https://doi.org/10.1371/journal.pdig.0000135)

Ng MY, Youssef A, Pillai M, Shah V, Hernandez-Boussard T. Scaling equitable artificial intelligence in healthcare with machine learning operations. *BMJ Health Care Inform*. 2024;31(1). doi:[10.1136/bmjhci-2024-101101](https://doi.org/10.1136/bmjhci-2024-101101)

Ngwa W, Pressley A, Wilson VM, et al. A comprehensive cancer center in the cloud powered by AI can reduce health disparities. *Nat Med*. 2024;30(9):2388-2389. doi:[10.1038/s41591-024-03119-y](https://doi.org/10.1038/s41591-024-03119-y)

O’Connor MI. Equity360: Gender, Race, and Ethnicity-The Power of AI to Improve or Worsen Health Disparities. *Clin Orthop Relat Res*. 2024;482(4):591-594. doi:[10.1097/CORR.0000000000002986](https://doi.org/10.1097/CORR.0000000000002986)

O’Connor S, Booth RG. Algorithmic bias in health care: Opportunities for nurses to improve equality in the age of artificial intelligence. *Nurs Outlook*. 2022;70(6):780-782. doi:[10.1016/j.outlook.2022.09.003](https://doi.org/10.1016/j.outlook.2022.09.003)

Okenyi E, Walker L. Advantages and challenges of AI in enhancing healthcare equity. *Prescriber*. 2024;35(1):5-8.

Olawade DB, Bolarinwa OA, Adebisi YA, Shongwe S. The role of artificial intelligence in enhancing healthcare for people with disabilities. *Social Science & Medicine*. 2025;364:N.PAG-N.PAG.

Paik KE, Hicklen R, Kaggwa F, et al. Digital Determinants of Health: Health data poverty amplifies existing health disparities-A scoping review. *PLOS Digit Health*. 2023;2(10):e0000313. doi:[10.1371/journal.pdig.0000313](https://doi.org/10.1371/journal.pdig.0000313)

Park JI. Harnessing Artificial Intelligence to Promote Health Equity. *Clin Nurs Res*. 2024;33(7):507-508. doi:[10.1177/10547738241270546](https://doi.org/10.1177/10547738241270546)

Paulus JK, Kent DM. Predictably unequal: understanding and addressing concerns that algorithmic clinical prediction may increase health disparities. *NPJ Digit Med*. 2020;3:99. doi:[10.1038/s41746-020-0304-9](https://doi.org/10.1038/s41746-020-0304-9)

Polevikov S. Advancing AI in healthcare: A comprehensive review of best practices. *Clinica Chimica Acta*. 2023;548:N.PAG-N.PAG.

Prathomwong P, Singsuriya P. Ethical framework of digital technology, artificial intelligence, and health equity. *Asia Social Issues*. 2022;15(5):252136-252136.

Rajkomar A, Hardt M, Howell MD, Corrado G, Chin MH. Ensuring Fairness in Machine Learning to Advance Health Equity. *Ann Intern Med*. 2018;169(12):866-872. doi:[10.7326/M18-1990](https://doi.org/10.7326/M18-1990)

Ranasinghe ND. Addressing Healthcare Disparities with Computer Vision and AI: Investigating the Potential of Intelligent Systems to Improve Access, Equity, and Outcomes in Underserved Populations. *Journal of Applied Smart HealthCare Informatics*. 2023;13(12):21-30.

Raza MM, Venkatesh KP, Kvedar JC. Promoting racial equity in digital health: applying a cross-disciplinary equity framework. *NPJ Digit Med*. 2023;6(1):3. doi:[10.1038/s41746-023-00747-5](https://doi.org/10.1038/s41746-023-00747-5)

Raza S, Shaban-Nejad A, Dolatabadi E, Mamiya H. Exploring Bias and Prediction Metrics to Characterise the Fairness of Machine Learning for Equity-Centered Public Health Decision-Making: A Narrative Review. *IEEE ACCESS*. 2024;12:180815-180829. doi:[10.1109/ACCESS.2024.3509353](https://doi.org/10.1109/ACCESS.2024.3509353)

Rinderknecht F, Nwandu L, Daneshjou R, Lester J. The Impact of Artificial Intelligence on Health Equity in Dermatology (JUL,10.1007/s13671-024-00436-w, 2024). *CURRENT DERMATOLOGY REPORTS*. 2024;13(3):156-156. doi:[10.1007/s13671-024-00438-8](https://doi.org/10.1007/s13671-024-00438-8)

Ritoré Á, Jiménez CM, González JL, et al. The role of Open Access Data in democratizing healthcare AI: A pathway to research enhancement, patient well-being and treatment equity in Andalusia, Spain. *PLOS Digit Health*. 2024;3(9):e0000599. doi:[10.1371/journal.pdig.0000599](https://doi.org/10.1371/journal.pdig.0000599)

Robinson WR, Renson A, Naimi AI. Teaching yourself about structural racism will improve your machine learning. *Biostatistics*. 2020;21(2):339-344. doi:[10.1093/biostatistics/kxz040](https://doi.org/10.1093/biostatistics/kxz040)

Rodriguez JA, Alsentzer E, Bates DW. Leveraging large language models to foster equity in healthcare. *J Am Med Inform Assoc*. 2024;31(9):2147-2150. doi:[10.1093/jamia/ocae055](https://doi.org/10.1093/jamia/ocae055)

Röösli E, Rice B, Hernandez-Boussard T. Bias at warp speed: how AI may contribute to the disparities gap in the time of COVID-19. *J Am Med Inform Assoc*. 2021;28(1):190-192. doi:[10.1093/jamia/ocaa210](https://doi.org/10.1093/jamia/ocaa210)

Ross P. Racial Equity Implications of Artificial Intelligence in Health Care. *Food and Drug Law Journal*. 2022;77(2):265-273.

Rothlisberger PN. AI-Powered Patient-Centered Care: A Call to Action for Innovation. *Journal of Healthcare Management*. 2024;69(4):255-266.

Samorani M, Blount LG. Machine Learning and Medical Appointment Scheduling: Creating and Perpetuating Inequalities in Access to Health Care. *Am J Public Health*. 2020;110(4):440-441. doi:[10.2105/AJPH.2020.305570](https://doi.org/10.2105/AJPH.2020.305570)

Sendak MP, Kim JY, Hasan A, et al. Empowering US healthcare delivery organizations: Cultivating a community of practice to harness AI and advance health equity. *PLOS Digit Health*. 2024;3(6):e0000513. doi:[10.1371/journal.pdig.0000513](https://doi.org/10.1371/journal.pdig.0000513)

Sharma R, Anderson T. The Promise and Peril of Advancing Health Equity through Artificial Intelligence. *Health Lawyer*. 2023;35(6):23-65.

Shiwani T, Relton S, Evans R, et al. New Horizons in artificial intelligence in the healthcare of older people. *Age & Ageing*. 2023;52(12):1-11.

Sikstrom L, Maslej MM, Hui K, Findlay Z, Buchman DZ, Hill SL. Conceptualising fairness: three pillars for medical algorithms and health equity. *BMJ Health Care Inform*. 2022;29(1). doi:[10.1136/bmjhci-2021-100459](https://doi.org/10.1136/bmjhci-2021-100459)

Skorburg JA, Yam J. Is There an App for That?: Ethical Issues in the Digital Mental Health Response to COVID-19. *AJOB Neuroscience*. 2022;13(3):177-190.

Smith MJ, Axler R, Bean S, Rudzicz F, Shaw J. Four equity considerations for the use of artificial intelligence in public health. *Bull World Health Organ*. 2020;98(4):290-292. doi:[10.2471/BLT.19.237503](https://doi.org/10.2471/BLT.19.237503)

Sorin V, Klang E. Artificial Intelligence and Health Care Disparities in Radiology. *Radiology*. 2021;301(3):E443. doi:[10.1148/radiol.2021210566](https://doi.org/10.1148/radiol.2021210566)

Souza R, Stanley E, Forkert N. On the Relationship Between Open Science in Artificial Intelligence for Medical Imaging and Global Health Equity. In: Vol 14242. 2023:289-300. doi:[10.1007/978-3-031-45249-9_28](https://doi.org/10.1007/978-3-031-45249-9_28)

Straw I. The automation of bias in medical Artificial Intelligence (AI): Decoding the past to create a better future. *Artificial Intelligence in Medicine*. 2020;110:N.PAG-N.PAG.

Stypińska J, Franke A. AI revolution in healthcare and medicine and the (re-)emergence of inequalities and disadvantages for ageing population. *Front Sociol*. 2022;7:1038854. doi:[10.3389/fsoc.2022.1038854](https://doi.org/10.3389/fsoc.2022.1038854)

Takshi S. Unexpected Inequality: Disparate-Impact From Artificial Intelligence in Healthcare Decisions. *J Law Health*. 2021;34(2):215-251.

Tat E, Bhatt DL, Rabbat MG. Addressing bias: artificial intelligence in cardiovascular medicine. *Lancet Digit Health*. 2020;2(12):e635-e636. doi:[10.1016/S2589-7500(20)30249-1](https://doi.org/10.1016/S2589-7500(20)30249-1)

Thamman R, Yong CM, Tran AH, Tobb K, Brandt EJ. Role of Artificial Intelligence in Cardiovascular Health Disparities: The Risk of Greasing the Slippery Slope. *JACC Adv*. 2023;2(7):100578. doi:[10.1016/j.jacadv.2023.100578](https://doi.org/10.1016/j.jacadv.2023.100578)

Thomasian NM, Eickhoff C, Adashi EY. Advancing health equity with artificial intelligence. *J Public Health Policy*. 2021;42(4):602-611. doi:[10.1057/s41271-021-00319-5](https://doi.org/10.1057/s41271-021-00319-5)

Timmons AC, Duong JB, Simo Fiallo N, et al. A Call to Action on Assessing and Mitigating Bias in Artificial Intelligence Applications for Mental Health. *Perspectives on Psychological Science*. 2023;18(5):1062-1096.

Uygun İLi̇Khan S, Özer M, Tanberkan H, Bozkurt V. How to mitigate the risks of deployment of artificial intelligence in medicine? *Turkish Journal of Medical Sciences*. 2024;54(3):483-492.

van Kolfschooten H. The AI cycle of health inequity and digital ageism: mitigating biases through the EU regulatory framework on medical devices. *J Law Biosci*. 2023;10(2):lsad031. doi:[10.1093/jlb/lsad031](https://doi.org/10.1093/jlb/lsad031)

Wawira Gichoya J, McCoy LG, Celi LA, Ghassemi M. Equity in essence: a call for operationalising fairness in machine learning for healthcare. *BMJ Health Care Inform*. 2021;28(1). doi:[10.1136/bmjhci-2020-100289](https://doi.org/10.1136/bmjhci-2020-100289)

Webb J. Healthcare Resource Allocation, Machine Learning, and Distributive Justice. *American Philosophical Quarterly*. 2025;62(1):33-52.

Webster P, Neal KR. What’s artificial intelligence (AI) got to do with it-inequality and public health? *J Public Health (Oxf)*. 2024;46(2):207-208. doi:[10.1093/pubmed/fdae052](https://doi.org/10.1093/pubmed/fdae052)

Wesson P, Hswen Y, Valdes G, Stojanovski K, Handley MA. Risks and Opportunities to Ensure Equity in the Application of Big Data Research in Public Health. *Annu Rev Public Health*. 2022;43:59-78. doi:[10.1146/annurev-publhealth-051920-110928](https://doi.org/10.1146/annurev-publhealth-051920-110928)

Wu B, Zhu Z. Multiple approaches to advance health equity in nursing science: Recruitment, data, and dissemination. *NURSING OUTLOOK*. 2025;73(1). doi:[10.1016/j.outlook.2024.102343](https://doi.org/10.1016/j.outlook.2024.102343)

Wu Y, Liu XM. Navigating the Ethical Landscape of AI in Healthcare: Insights From a Content Analysis. *IEEE Technology & Society Magazine*. 2023;42(3):76-87.

Ye H. Address health inequities among human beings is an ethical matter of urgency, whether or not to develop more powerful AI. *J Med Ethics*. 2024;50(12):820-821. doi:[10.1136/jme-2024-110171](https://doi.org/10.1136/jme-2024-110171)

Yu L, Zhai X. Use of artificial intelligence to address health disparities in low- and middle-income countries: a thematic analysis of ethical issues. *Public Health*. 2024;234:77-83. doi:[10.1016/j.puhe.2024.05.029](https://doi.org/10.1016/j.puhe.2024.05.029)

Zheng Y, Wu Y, Feng B, Wang L, Kang K, Zhao A. Enhancing Diabetes Self-management and Education: A Critical Analysis of ChatGPT’s Role. *Ann Biomed Eng*. 2024;52(4):741-744. doi:[10.1007/s10439-023-03317-8](https://doi.org/10.1007/s10439-023-03317-8)

Zidaru T, Morrow EM, Stockley R. Ensuring patient and public involvement in the transition to AI-assisted mental health care: A systematic scoping review and agenda for design justice. *Health Expect*. 2021;24(4):1072-1124. doi:[10.1111/hex.13299](https://doi.org/10.1111/hex.13299)
